# Description of Kilburn Wells, and Analysis of Their Water

**Published:** 1794

**Authors:** Joh. Godfr. Schmeisser


XII. *Deſcription of Kilburn Wells, and Analyſis of their Water. By Mr.* Joh. Godfr. Schmeiſſer. Vide *Philoſophical Tranſactions of the Royal Society of London, for the Year* 1792. *Part* I. 4to. London, 1792.

THESE wells lie in a meadow, to the right of the Edgeware road, about two miles from London.

The author obſerves that they ſpring about twelve feet below the ſurface; that the water is not not perfectly bright, but of rather a milky hue; that it has a mild and bitteriſh taſte, with little or no briſkneſs, as containing a very ſmall proportion of fixed air; that on dipping for it, or otherwiſe agitating it, a ſulphureous ſmell is perceived near the ſurface, which, however, ſoon goes off in a temperature of 80° of Fahrenheit's thermometer; and that the changes in the atmoſphere do not appear to affect either the quantity or quality of the water.

Mr. Schmeiſſer found the ſpecific gravity of the Kilburn water to be to diſtilled water as 1,0071 : 1,0000; and its general temperature 53°, which was not affected by a change of ten degrees in the temperature of the atmoſphere.

While the water continued at reſt, no ebullition of fixed air, we are told, was perceived, and ſcarce any ſulphureous ſmell.

That this mineral water ſo eaſily parts with the hepatic air (perceivable on agitating it) if it be ſhaken in a warmer temperature, or tranſported from one place to another, is, our author thinks, probably owing to the fixed air which it contains; for as this aërial acid has a great affinity to phlogiſton, ſo, he obſerves, it may hence be inferred, that fixed and hepatic air cannot exiſt together in a mineral water, but that the latter will be deſtroyed, as the fixed air is developed by gentle warmth.

For the detail of Mr. Schmeiſſer's ingenious experiments on this water, we muſt refer the chemical reader to the work itſelf, as they cannot well be abridged. They conſiſt of experiments with reagent ſubſtances; of experiments to aſcertain the properties and proportion of the elaſtic fluids contained in the water; and, laſtly, of experiments to aſcertain the fixed conſtituent parts of the water, and their properties.

The reſults of this ſeemingly very accurate analyſis are included in the following ſummary of the conſtituent parts of the Kilburn water, in 24 pounds.

| | | | | | |
|---|---|---|---|---|---|
| Fixed air - - | | 84 | cubic inches | | |
| Hepatic air - | near | 36 | | | |
| Vitriolated magneſia - | | 910 | grains, equal to ℥ij ʒiiss, Apothecary's weight. | | |
| Vitriolated natron - | | 282 | gr. | = | ʒv. lij grains |
| Muriated natron - | | 60 | gr. | = | 75 gr. |
| Selenite - - | | 130 | gr. | = | ʒij xlij gr. |
| Muriated magneſia - | | 128 | gr. | = | ʒij xl gr. |
| ——— calcareous earth | | 6 | gr. | = | 7½ gr. |
| Aerated magneſia - | | 12½ | gr. | = | 15 gr. |
| ——— calcareous earth | | 24 | gr. | = | 30 gr. |
| Calx of iron - | | 3⅛ | gr. | = | 4 gr. |
| Reſinous matter - | | 6 | gr. | = | 7½ gr. |

Sum 1561⅝ grains, equal to medicinal weight, 4 ounces, 0 drams, and 32 grains.

XIII. *An*

